# Development of Expanded Takayanagi Model for Tensile Modulus of Carbon Nanotubes Reinforced Nanocomposites Assuming Interphase Regions Surrounding the Dispersed and Networked Nanoparticles

**DOI:** 10.3390/polym12010233

**Published:** 2020-01-17

**Authors:** Yasser Zare, Kyong Yop Rhee

**Affiliations:** Department of Mechanical Engineering, College of Engineering, Kyung Hee University, Yongin 446-701, Korea; y.zare@aut.ac.ir

**Keywords:** polymer CNT nanocomposites, filler network, interphase, tensile modulus, modeling

## Abstract

In this paper, we consider the interphase regions surrounding the dispersed and networked carbon nanotubes (CNT) to develop and simplify the expanded Takayanagi model for tensile modulus of polymer CNT nanocomposites (PCNT). The moduli and volume fractions of dispersed and networked CNT and the surrounding interphase regions are considered. Since the modulus of interphase region around the dispersed CNT insignificantly changes the modulus of nanocomposites, this parameter is removed from the developed model. The developed model shows acceptable agreement with the experimental results of several samples. “*E*_R_” as nanocomposite modulus per the modulus of neat matrix changes from 1.4 to 7.7 at dissimilar levels of “*f*” (CNT fraction in the network) and network modulus. Moreover, the lowest relative modulus of 2.2 is observed at the smallest levels of interphase volume fraction ($\phi_{i}$ < 0.017), while the highest “$\phi_{i}$” as 0.07 obtains the highest relative modulus of 11.8. Also, the variation of CNT size (radius and length) significantly changes the relative modulus from 2 to 20.

## 1. Introduction

Carbon nanotubes (CNT) have involved significant interest in nanocomposites due to amazing mechanical behavior and good electrical conductivity, as well as nanometer size and high aspect ratio [1,2,3,4,5,6,7,8,9,10,11,12,13,14,15,16]. It was reported that only a low content of CNT is enough to prepare a nanocomposite with significant mechanical and conductivity properties. However, polymer CNT nanocomposites (PCNT) have been faced with two main problems. A poor dispersion of CNT is commonly shown in the previous reports, due to the instinct nano-size of CNT, which produces bundles and ropes [17,18]. Also, the weak interfacial bonding, due to the smooth surface of CNT limits the load transfer from matrix to CNT [19,20]. Many researchers have attempted to overcome these obstacles by chemical functionalization of CNT, which can provide functional groups on the CNT surface and form the covalent bonding with the matrix [21,22].

The electrical conductivity of PCNT significantly increases when the CNT concentration reaches the percolation threshold, because the percolation threshold is the minimum volume fraction of nanoparticles creating the network in the nanocomposites [23,24,25]. After the percolation threshold, conductivity of PCNT increases with CNT addition. Also, the substantial modulus and impact toughness of polymer nanocomposites were also attributed to the formation of a strong network of filler above percolation threshold which is called mechanical percolation [26,27]. Favier et al. [28], as pioneers, have discussed the percolation effect for the remarkable improvement of shear modulus in the films reinforced with cellulose whiskers. However, the mechanical percolation in polymer nanocomposite has received restricted focus despite its important role.

The large surface area per volume of nanoparticles as a main advantage and the strong interaction between polymer matrix and nanofiller create a third phase as interphase in polymer nanocomposites, which is different from other phases [29,30,31,32,33]. The interphase has shown a main role in the effective properties of polymer nanocomposites. It was studied in the preceding works that the interphase can highly enhance the mechanical behavior of nanocomposites [34,35]. So, the interphase region demonstrates a reinforcing role, which causes the conventional models to underestimate the modulus.

The big interphase/interfacial area may form a percolated network at lower concentration of nanoparticles due to the connection of interphase region as pseudo-percolation [36,37]. This approach was confirmed by theoretical and experimental results in polymer nanocomposites. For example, Celzard et al. [38] related the difference between the measured and the predicted percolation threshold to the interfacial interaction between polymer matrix and particles. In fact, the pseudo-percolation accelerates the percolation in nanocomposites. Nevertheless, this subject has been tersely investigated in the former studies.

Loos and Manas-Zloczower [39] expanded the Takayanagi model to two forms assuming the networking and dispersion of nanoparticles in PCNT above percolation threshold. In form 1, the percolation threshold causes a negative effect on the modulus of nanocomposites and the filler volume fractions above percolation threshold decrease the reinforcement of samples, because it assumes that the dispersed nanoparticles bear a higher load compared to the matrix. However, form 2 accepts that the filler network supports much higher load than the matrix, which is reasonable for polymer nanocomposites above the glass transition temperature of polymer matrix. Therefore, the expanded model according to form 2 suggests the reinforcing efficiency of networked nanoparticles above percolation threshold, which is developed in our present paper. The expanded model (form 2) does not consider the interphase regions around the dispersed and networked nanoparticles neglecting the important reinforcing and percolating effects of interphase zone in the modulus. In our present study, the interphase role develops the expanded model by Loos and Manas-Zloczower for tensile modulus of PCNT. Moreover, the fraction of nanoparticles in the network and the mechanical percolation threshold are correlated to the radius and length of CNT and thickness of interphase. The developed model and equations are applied to predict the tensile modulus, interphase properties, and percolation threshold in some samples. Similarly, the effects of all CNT, network and interphase parameters on the predicted modulus are approved. The main novelty of this paper is the development of a simple and applicable model for modulus of PCNT considering the properties of all components such as the dispersed and networked CNT, as well as the surrounding interphase regions. Additionally, the developed model includes the simple, meaningful, and applicable parameters for prediction of modulus, while the previous models usually disregarded the mechanical percolation, interphase region, and CNT network, or included the complex and baffling parameters.

## 2. Equations

In this section, we consider the interphase regions around the dispersed and networked CNT to develop the expanded Takayanagi model.

The expanded Takayanagi model by Loos and Manas-Zloczower [39] reflected the reinforcing efficiency of filler network (form 2) as:

(1) $$E=\frac{\phi_{N}(1-\phi_{f})E_{d}^{}E_{N}^{}+\phi_{N}(\phi_{f}-\phi_{N})E_{m}^{}E_{N}^{}+{\left(1-\phi_{N}\right)}^{2}E_{d}^{}E_{m}^{}}{(1-\phi_{f})E_{d}^{}+(\phi_{f}-\phi_{N})E_{m}^{}}$$

where “*E*” is the tensile modulus of nanocomposites, which is measured by tensile test. “$\phi_{f}$” and “$\phi_{N}$” are the volume fractions of nanofiller and networked particles, respectively. Also, “*E*_d_”, “*E*_N_” and “*E*_m_” are the tensile moduli of dispersed nanofiller, filler network, and polymer matrix, respectively. Both “$\phi_{f}$” and “$\phi_{N}$” can be added up to 1. Equation (1) does not consider a particulate filler shape, but we develop it for the modulus of only polymer CNT nanocomposites in the current study.

The absence of network ($\phi_{N}$ = 0) reduces Equation (1) to series model [22] as:

(2) $$E=\frac{E_{d}^{}E_{m}^{}}{(1-\phi_{f})E_{d}^{}+\phi_{f}E_{m}^{}}$$

Now, the interphase regions around the dispersed and networked nanoparticles are assumed to develop Equation (1). The volume fractions and moduli of the interphase regions around the dispersed and networked CNT develop Equation (1) to:

(3) $$\begin{array}{l} E=\frac{(\phi_{N}+f\phi_{i})(1-\phi_{f}-\phi_{i})(E_{d}^{}+E_{id})(E_{N}^{}+E_{iN})\ldots }{(1-\phi_{f}-\phi_{i})(E_{d}+E_{id}^{})\ldots }\\ \frac{\ldots +(\phi_{N}+f\phi_{i})(\phi_{f}+\phi_{i}-\phi_{N}-f\phi_{i})(E_{N}^{}+E_{iN})E_{m}^{}+{\left(1-\phi_{N}-f\phi_{i}\right)}^{2}(E_{d}^{}+E_{id})E_{m}^{}}{\ldots +(\phi_{f}+\phi_{i}-\phi_{N}-f\phi_{i})E_{m}^{}} \end{array}$$

where “*f*” is the fraction of CNT establishing the network and “$\phi_{i}$” is the total volume fraction of interphase region in PCNT. Similarly, “*E*_id_” and “*E*_iN_” show the moduli of interphases around the dispersed and networked nanoparticles, respectively. The subscripts “N” and “d” denote the networked and dispersed CNT, respectively, while the subscripts “iN” and “id” show the interphase regions surrounding the networked and dispersed CNT, respectively. Additionally, “$\phi_{f}$”, “$\phi_{N}$” and “$\phi_{i}$” can be increased up to 1.

Now, we present the simple equations for “*f*”, “$\phi_{N}$” and “$\phi_{i}$”.

The percolation threshold in PCNT was expressed [40] as:

(4) $$\phi_{p}=\frac{\pi R^{2}l+(4/3)\pi R^{3}}{\frac{32}{3}\pi {\left(R+t\right)}^{3}[1+\frac{3}{4}(\frac{l}{R+t})+\frac{3}{32}{\left(\frac{l}{R+t}\right)}^{2}]}$$

where “*R*” and “*l*” are the radius and length of CNT and “*t*” is interphase thickness.

Also, “$\phi_{i}$” as the total volume fraction of interphase regions in PCNT is determined [41] by:

(5) $$\phi_{i}=\phi_{f}{\left(1+\frac{t}{R}\right)}^{2}-\phi_{f}$$

The effective volume fraction of CNT in PCNT assumes the volume fractions of CNT and surrounding interphase [41] as:

(6) $$\phi_{eff}=\phi_{f}+\phi_{i}=\phi_{f}{\left(1+\frac{t}{R}\right)}^{2}$$

Now, the fraction of CNT, which forms the network in PCNT after percolation threshold is suggested [41] as:

(7) $$f=\frac{\phi_{eff}^{1/3}-\phi_{p}^{1/3}}{1-\phi_{p}^{1/3}}$$

When “$\phi_{p}$” (Equation (4)) and “$\phi_{eff}$” (Equation (6)) are replaced into above equation, “*f*” is calculated by CNT concentration, CNT size and interphase thickness.

The volume fraction of CNT network in nanocomposite is calculated by:

(8) $$\phi_{N}=\frac{f\phi_{f}}{1-(1-f)\phi_{f}}≅f\phi_{f}$$

Assuming the above equation into Equation (3) results in:

(9) $$\begin{array}{l} E=\frac{(f\phi_{f}+f\phi_{i})(1-\phi_{f}-\phi_{i})(E_{d}^{}+E_{id})(E_{N}^{}+E_{iN})\ldots }{(1-\phi_{f}-\phi_{i})(E_{d}+E_{id}^{})\ldots }\\ \frac{\ldots +(f\phi_{f}+f\phi_{i})(\phi_{f}+\phi_{i}-f\phi_{f}-f\phi_{i})(E_{N}^{}+E_{iN})E_{m}^{}+{\left(1-f\phi_{f}-f\phi_{i}\right)}^{2}(E_{d}^{}+E_{id})E_{m}^{}}{\ldots +(\phi_{f}+\phi_{i}-f\phi_{f}-f\phi_{i})E_{m}^{}} \end{array}$$

The relative modulus of nanocomposites is defined by dividing of “*E*” to matrix modulus as:

(10) $$\begin{array}{l} E_{R}=\frac{(f\phi_{f}+f\phi_{i})(1-\phi_{f}-\phi_{i})(E_{d}^{}+E_{id})(E_{N}^{}+E_{iN})/E_{m}\ldots }{(1-\phi_{f}-\phi_{i})(E_{d}+E_{id}^{})\ldots }\\ \frac{\ldots +(f\phi_{f}+f\phi_{i})(\phi_{f}+\phi_{i}-f\phi_{f}-f\phi_{i})(E_{N}^{}+E_{iN})+{\left(1-f\phi_{f}-f\phi_{i}\right)}^{2}(E_{d}^{}+E_{id})}{\ldots +(\phi_{f}+\phi_{i}-f\phi_{f}-f\phi_{i})E_{m}^{}} \end{array}$$

When “*f*” (Equation (7)) and “$\phi_{i}$” (Equation (5)) are substituted into above equation, the relative modulus correlates to the main properties of CNT (dispersed and networked ones) and interphase regions surrounding the dispersed and networked CNT.

**Figure 1:**
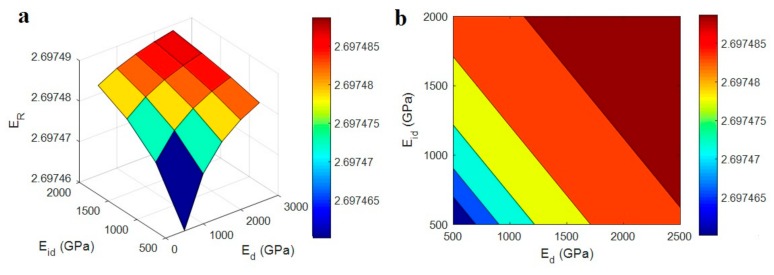
The influences of “*E*_d_” and “*E*_id_” parameters on the predicted modulus (Equation (10)): (**a**) 3D and (**b**) contour designs.

Figure 1 depicts the effects of “*E*_d_” and “*E*_id_” parameters on the relative modulus by Equation (10) at average $\phi_{f}$ = 0.01, *R* = 10 nm, *l* = 10 μm, *t* = 5 nm, *E*_N_ = 500 GPa and *E*_iN_ = 200 GPa. Although the positive effects of “*E*_d_” and “*E*_id_” on the modulus are clear, the different levels of these factors slightly change the modulus of nanocomposites. So, the modulus of interphase zone surrounding dispersed CNT can be neglected in the developed model, due to its insignificant effect on the modulus of nanocomposites.

It can be concluded that the network properties and the surrounding interphase play the main roles in the stiffness of CNT reinforced nanocomposites. This is expected, because the CNT network has more significant properties compared to the dispersed nanoparticles. Earlier studies have shown that the networks of nanofillers cause the noteworthy properties in the nanocomposites [42,43,44]. Furthermore, some terms can be simplified in Equation (10). The extremely low volume fractions of CNT and interphase region in PCNT simplify these terms: $\left(1-\phi_{f}-\phi_{i}\right)$ ≈ $\left(1-f\phi_{f}-f\phi_{i}\right)$ ≈ 1.

Removing of “*E*_id_” and considering the mentioned simplifications develop Equation (10) to:

(11) $$E_{R}=\frac{f(\phi_{f}+\phi_{i})E_{d}^{}(E_{N}^{}+E_{iN})/E_{m}^{}+f(\phi_{f}+\phi_{i})(\phi_{f}+\phi_{i}-f\phi_{f}-f\phi_{i})(E_{N}^{}+E_{iN})+E_{d}^{}}{E_{d}+(\phi_{f}+\phi_{i}-f\phi_{f}-f\phi_{i})E_{m}^{}}$$

which considers the properties of CNT, CNT network, and the interphase zone surrounding the network in the modulus of PCNT. Since this model only reflects the reinforcing of nanocomposites as CNT concentration increases, Equation (11) is not applicable when the addition of CNT does not promote the modulus of nanocomposites.

## 3. Results and Discussion

### 3.1. Evaluation of Model by Experimental Data

Four samples from literature including chitosan/multi-walled carbon nanotubes (MWCNT) (*E*_m_ = 2 GPa, *R* = 8 nm and *l* = 7.5 μm) [45], PP/MWCNT (*E*_m_ = 0.77 GPa, *R* = 8 nm and *l* = 10 μm) [46], PVA/MWCNT (*E*_m_ = 1.95 GPa, *R* = 10 nm and *l* = 10 μm) [47] and epoxy/MWCNT (*E*_m_ = 0.52 GPa, *R* = 25 nm and *l* = 10 μm) [17] are considered. The properties of polymer matrix (tensile modulus) and CNT (CNT size) were reported in the original references. Also, “*E*_d_” is considered 1000 GPa [48]. However, the values of interphase properties surrounding the networked CNT and network modulus are calculated by fitting the experimental results of modulus to the developed model. Since the developed model suggests reasonable levels for the interphase properties and network modulus, these calculations are defensible.

**Figure 2:**
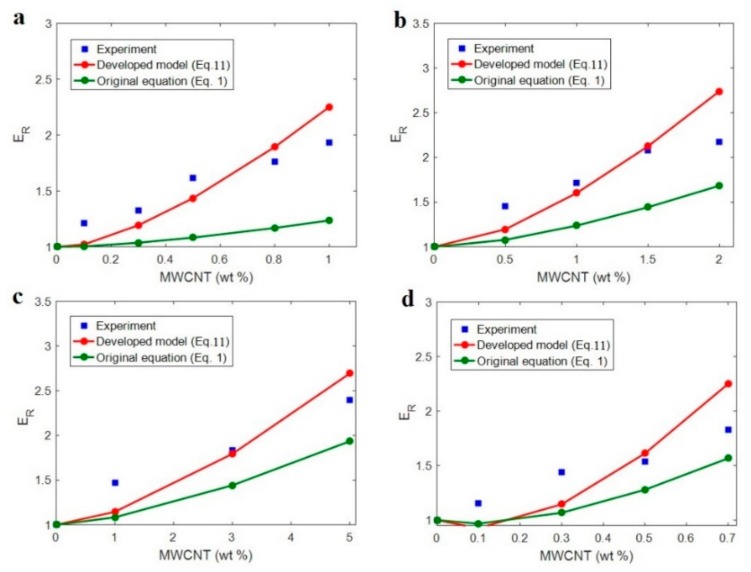
The experimental data of relative modulus and the calculations of original and developed models for (**a**) chitosan/MWCNT [45], (**b**) PP/MWCNT [46], (**c**) PVA/MWCNT [47] and (**d**) epoxy/MWCNT [17] samples.

Figure 2 compares the experimental results of relative modulus with the calculations of original (Equation (1)) and developed (Equation (11)) models for all samples. The original model cannot fit to the experimental data and underestimates the modulus, but the experimental data are well fitted to the developed model demonstrating the acceptable calculations of the model for the present samples. From this fitting, the different levels for the interphase and network properties are calculated for each sample. We report the average values of these parameters for the samples. The average values of (*t*, *E*_iN_, *E*_N_) are obtained as (3.5 ± 0.3, 70 ± 10, 300 ± 40), (3 ± 0.3, 100 ± 15, 280 ± 35), (2 ± 0.2, 50 ± 4, 180 ± 25) and (6 ± 1, 400 ± 70, 900 ± 70) (nm, GPa, GPa) for chitosan/MWCNT, PP/MWCNT, PVA/MWCNT and epoxy/MWCNT samples, respectively. These results are meaningful, because they change in the reasonable ranges. They demonstrate the thickest and the strongest interphase in epoxy/MWCNT sample, while PVA/MWCNT shows the thinnest and the poorest interphase region surrounding the networked CNT. Moreover, the strongest and the poorest networks are shown in epoxy/MWCNT and PVA/MWCNT samples, respectively. These calculations are expected, because the strong interfacial adhesion between epoxy matrix and functionalized CNT produces the thick and strong interphase in this sample [17], while the weak interfacial bonding between PVA and CNT causes the poor interphase for this sample.

### 3.2. Parametric Analyses

**Figure 3:**
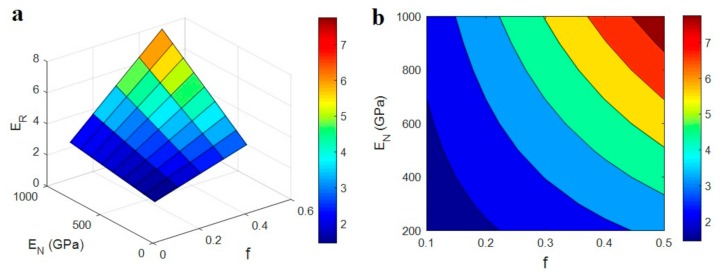
The effects of “*f*” and “*E*_N_” parameters on the relative modulus (Equation (11)): (**a**) 3D and (**b**) contour plots.

The influences of all parameters on the predicted modulus (Equation (11)) are also plotted and discussed. Figure 3 shows the calculations of relative modulus at different “*f*” and “*E*_N_” levels and average $\phi_{f}$ = 0.01, *R* = 10 nm, *l* = 10 μm, *t* = 5 nm, *E*_d_ = 1000 GPa, *E*_id_ = 200 GPa and *E*_iN_ = 500 GPa. The relative modulus changes from 1.4 to about 7.7 by variation of “*f*” and “*E*_N_” parameters, which reveals a wide range. The highest “*E*_R_” is obtained as about 7.7 at maximum *f* = 0.5 and *E*_N_ = 1000 GPa. Furthermore, the least relative modulus of 1.4 is reported at minimum *f* = 0.1 and *E*_N_ = 200 GPa demonstrating the direct influences of these parameters on the modulus. It means that the higher levels of both “*f*” and “*E*_N_” parameters produce stiffer PCNT.

“*f*” as the fraction of CNT in the network shows the reasonable effect on the modulus. A higher number of CNT in the network undoubtedly make a denser network, which can bear a higher level of stress [49]. Therefore, it is sensible to obtain a higher modulus by a larger number of CNT in the network. The literature reports also revealed a direct relation between the modulus of nanocomposites and the density of network [50]. Also, a stronger network can prevent the deformation of sample when a high capacity load is applied. In fact, the advantage of networked nanoparticles over the dispersed ones is the connection of nanoparticles, which can bear a high level of stress. As a result, a dense and strong network results in the firm PCNT, which confirms predictions of the model.

**Figure 4:**
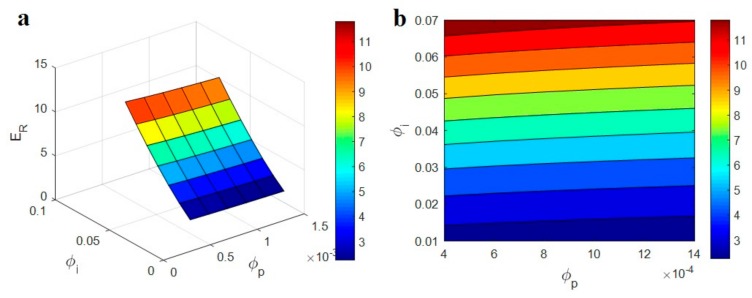
(**a**) 3D and (**b**) contour plots for the effects of “$\phi_{p}$ ” and “$\phi_{i}$” parameters on the relative modulus.

Figure 4 exhibits the roles of “$\phi_{p}$” and “$\phi_{i}$” parameters in the relative modulus at average $\phi_{f}$ = 0.01, *E*_m_ = 2 GPa, *E*_d_ = 1000 GPa, *E*_N_ = 500 GPa and *E*_iN_ = 200 GPa. The lowest relative modulus as 2.2 is observed at the smallest range of “$\phi_{i}$”, while the highest relative modulus as 11.8 is obtained by the highest “$\phi_{i}$”. Accordingly, “$\phi_{i}$” shows the positive influence on the modulus of PCNT based on the present model, while “$\phi_{p}$” is ineffective. Obviously, a low level of “$\phi_{p}$” enables the low concentration of nanoparticles to create a network. However, very low levels of “$\phi_{p}$” cannot affect the “*f*” and network volume fraction. So, “$\phi_{p}$” cannot manage the modulus of nanocomposites.

A higher value of “$\phi_{i}$” can play a significant effect on the modulus of PCNT. A higher level of “$\phi_{i}$” shows the formation of a thicker interphase (Equation (5)), which can considerably develop the modulus. In fact, both reinforcing and percolating roles of interphase region in PCNT directly relate to “$\phi_{i}$”. The first role is linked to the better properties of the interphase zone than those of the polymer matrix. A high “$\phi_{i}$” shows that a high volume of the sample is occupied by interphase, which reinforces the nanocomposite. Moreover, the percolating effect of interphase is correlated to the connectivity of the interphase region in PCNT. Clearly, a high level of “$\phi_{i}$” creates a quicker and better connectivity among the interphase area, which mainly stiffs the nanocomposite. As a result, the developed model correctly demonstrates the roles of “$\phi_{p}$” and “$\phi_{i}$” in the relative modulus.

**Figure 5:**
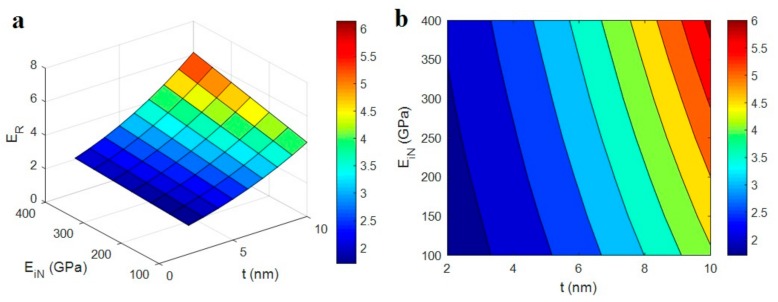
The roles of “*t*” and “*E*_iN_” parameters in the relative modulus according to Equation (11): (**a**) 3D and (**b**) contour plots.

The relative modulus of PCNT as a function of “*t*” and “*E*_iN_” parameters at $\phi_{f}$ = 0.01, *E*_m_ = 2 GPa, *R* = 10 nm, *l* = 10 μm, *E*_d_ = 1000 GPa and *E*_N_ = 500 GPa is observed in Figure 5. The smallest “*E*R” as 1.7 is shown at *t* = 2 nm and *E*_iN_ < 340 GPa, while the highest relative modulus as 6 is obtained at the highest *t* = 10 nm and *E*_iN_ = 400 GPa. As a result, a thin and poor interphase decreases the modulus of nanocomposites, but a thick and strong interphase increases the nanocomposite modulus to the highest level. It is concluded that the modulus of PCNT directly depends on “*t*” and “*E*_iN_” parameters. From the reinforcing view, a thick interphase is derived from a strong interfacial interaction/adhesion between polymer matrix and nanoparticles, which effectively transfers the stress from polymer matrix to nanoparticles, thus improving the modulus [48,51].

Previous studies have also shown the direct relation between the interphase thickness and its reinforcing effect [52]. Therefore, a direct relation exists between the thickness and reinforcing effect of interphase. Additionally, a thick interphase accelerates the connection of interphase regions, which decreases the percolation point, as mentioned. In other words, a thick interphase increases the possibility of percolation, whereas a thin interphase cannot promote the percolating role, which slightly improves the modulus. Also, a stronger interphase between polymer matrix and CNT network can transfer more stress from polymer matrix to network, but a weak interphase may result in debonding or pull-out of nanoparticles from polymer matrix [53]. Similarly, the high modulus of interphase plays a high stiffening effect, because the interphase reinforces the nanocomposites. Accordingly, a strong interphase results in a high modulus in PCNT. So, the developed model shows the correct influences of interphase thickness and modulus on the modulus of PCNT.

**Figure 6:**
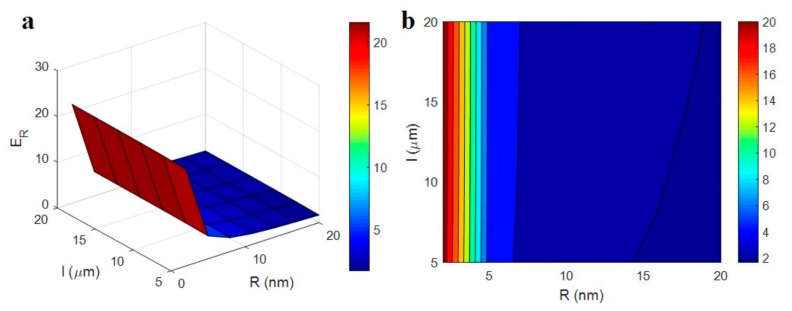
(**a**) 3D and (**b**) contour plots for the relative modulus as a function of “*R*” and “*l*” parameters.

Figure 6 displays the roles of “*R*” and “*l*” parameters in the relative modulus at $\phi_{f}$ = 0.01, *E*_m_ = 2 GPa, *t* = 5 nm, *E*_d_ = 1000 GPa, *E*_N_ = 500 GPa and *E*_iN_ = 200 GPa. The variation of “*R*” and “*l*” significantly changes the relative modulus from 2 to 20. As a result, the size of CNT extensively affects the tensile modulus of PCNT. This observation shows the most important role of CNT size in the modulus of nanocomposites, which should be considered in the future studies. The highest modulus is obtained by the thinnest and the longest CNT, while thick and short nanoparticles marginally improve the modulus.

It’s logical, because thin and long CNT promote the reinforcing efficiency of nanoparticles in PCNT. Thin and long CNT cause a high mechanical involvement between polymer matrix and nanofiller, which increases the modulus of nanocomposites [54]. In other words, the big interfacial area produced by thin and long CNT increases the polymer-nanoparticles connection, which raises the properties of polymer matrix. The literature reports have also shown the similar effects of filler size on the mechanical properties of polymer nanocomposites [55,56]. So, the developed model correctly predicts the effects of CNT dimensions on the modulus of PCNT.

## 4. Conclusions

The interphase regions around the dispersed and networked CNT were considered to develop and simplify the expanded Takayanagi model for tensile modulus of polymer nanocomposites above percolation threshold. We ignored the modulus of the interphase zone surrounding dispersed CNT, because it insignificantly changed the modulus of nanocomposites. The main advantages of the developed model in our present study are its simplicity and applicability for the tensile modulus of PCNT. Also, this model is a complete one, because it considers the main phases in the nanocomposites. Moreover, this paper considers the simple equations for mechanical percolation threshold and the fraction of networked CNT assuming the contribution of interphase region, while many studies disregarded the interphase regions and the networking of CNT after percolation threshold in nanocomposites. The developed model showed good fitting with the experimental data of several samples from literature, while the original model underestimated the modulus. Also, all parameters in the developed model acceptably handled the modulus of nanocomposites. The highest “*E*_R_” was obtained as 7.7 at maximum *f* = 0.5 and *E*_N_ = 1000 GPa, while the least relative modulus of 1.4 was calculated at minimum *f* = 0.1 and *E*_N_ = 200 GPa. So, both “*f*” and “*E*_N_” revealed the direct influences on the nanocomposite modulus. “$\phi_{i}$” also showed a positive influence on the modulus of PCNT, but “$\phi_{p}$” did not play a role. Additionally, the smallest “*E*_R_” as 1.7 was calculated at *t* = 2 nm and *E*_iN_ < 340 GPa, while the highest relative modulus of 6 was obtained at the highest levels of *t* = 10 nm and *E*_iN_ = 400 GPa. Therefore, thin and poor interphase significantly weakened the modulus of PCNT, but thick and strong interphase produced the strong nanocomposites. Finally, various CNT size significantly changed the relative modulus from 2 to 20, demonstrating that the dimensions of CNT extensively affect the tensile modulus of PCNT.
